# Supplementary material for: A phylogenomic study quantifies competing mechanisms for pseudogenization in prokaryotes—The Mycobacterium leprae case
Source: PLoS One. 2018 Nov 1;13(11):e0204322. doi: 10.1371/journal.pone.0204322 (PMC6211624; doi:10.1371/journal.pone.0204322)
Supplement: S1 Appendix — This file contains the supplementary text for this paper. (PDF) [file pone.0204322.s001.pdf]

Supplementary Text for "A Phylogenomic Study Quantifies Competing Mechanisms for Pseudogenization in Prokaryotes - the Mycobacterium leprae case"

This is a supplementary text to 'A Phylogenomic Study Quantifies Competing Mechanisms for Pseudogenization in Prokaryotes'. Specifically, we elaborate and provide background on phylogenetic tree and quartet trees in particular that are key to the main manuscript. We explain the synteny index that is the center in part two of the main manuscript. Finally, we describe the gene duplication verification procedure done in order to refute bogus low synteny.

1 Trees and Quartets

A phylogenetic tree T over a set of taxa set $\mathcal{X}$ is a tree whose leaves are labeled by $\mathcal{X}$. Removing an edge (or *branch*) from a tree creates two subtrees that naturally split $\mathcal{X}$. The split $(U, \mathcal{X} \setminus U)$ that is identified by an edge e is denoted by e_U or $e_{\mathcal{X} \setminus U}$ alternatively. Let T be an $\mathcal{X}$-tree and $\mathcal{X}'$ a subset of $\mathcal{X}$. Then the subtree of T that is *induced* by $\mathcal{X}'$ is obtained as follows: First, all the leaves in $\mathcal{X} \setminus \mathcal{X}'$, as well as paths leading exclusively to them, are removed. Next, all internal nodes with degree two are contracted. For two

trees T_1 and T_2, we say that T_1 and T_2 are the same if they contain the same set of splits. We say that T_1 *satisfies* T_2 if the subtree of T_1 that is induced by the leaves of T_2 is the same as T_2. Otherwise, T_2 is *violated* by T_1. For a set of trees $\mathcal{T} = \{T_1, \ldots, T_k\}$ with possibly overlapping leaves, we say that $\mathcal{T}$ is *consistent* (or *compatible*) if there exists a tree T^* over the union set of leaves of the trees in $\mathcal{T}$ that satisfies every tree $T_i \in \mathcal{T}$. Otherwise, $\mathcal{T}$ is *inconsistent* (or *incompatible*). The problem of finding such a consistent tree T^*, or a similar one if none exists, is known as the *supertree problem.* The tree T is *rooted* if all edges are directed away from a given node, the *root.* When edges are undirected, the tree is *unrooted.* The basic information unit in unrooted trees is a *quartet tree* (or simply a *quartet* when it is clear from the context), which is an unrooted tree that is defined over four taxa $\{a, b, c, d\}$ and is denoted by $a, b|c, d$ meaning that it has the split $(\{a, b\}, \{c, d\})$ (and this is the only *non-trivial* split in it, i.e., no singleton part). Specifically, a quartet $q = a, b|c, d$ is satisfied by a tree T if T has a split separating a, b from c, d (see Figure 1). A common special case of the supertree problem is when the input consists solely of quartets and the objective is to find a tree that satisfies the maximum number of quartets. This special case is denoted as the *Maximum Quartet Consistency* (MQC) problem. The general MQC problem is computationally intensive (exponential running time) but there are polynomial algorithms to solve special cases of it.

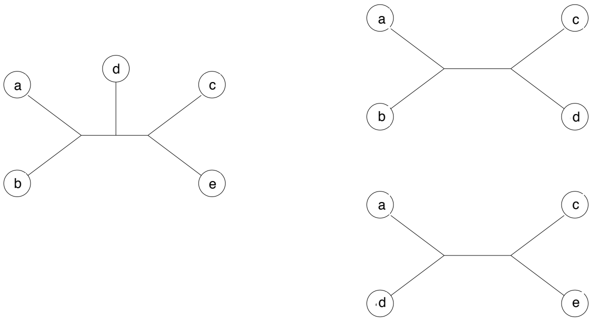

Figure 1: A toy example of quartets: In the right, two quartets are shown, $a, b|c, d$ and $a, d|c, e$. In the left, a tree over five taxa is shown. The tree *induces* the quartets $a, b|c, d$ and $a, d|c, e$ on the right. The topologies $a, c|b, d$ or $b, e|a, d$ (among others) are *not* induced by the larger tree on the left.

2 Synteny Index

We treat a genome as a sequence of genes (regardless of the DNA sequence of the gene, as this is beyond our resolution). The *k-neighborhood* of a gene in a genome is the set of genes at distance at most k from it along the genome (i.e. at most k genes upstream or downstream). The conservation of gene order between two genomes is called *synteny*. Consider a gene common to two genomes. Then, the *k-synteny index* (k-SI) of that gene, or just SI when it is clear from the context, is the number of common genes in the two k neighborhoods of this gene in both genomes. We remark that this definition extends naturally in cases of circular genomes where the k neighborhood should be taken accordingly (i.e. circularly). For the sake of completeness, if a gene is present only in one genome, we define its SI as zero. See Figure 2(a) for an illustration.

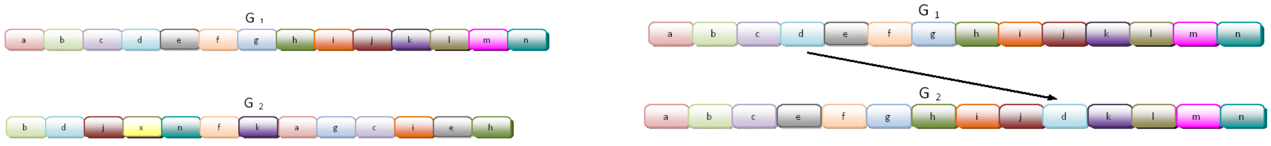

(a) (b)

Figure 2: (a) **Comparing G_1 with G_2 for $k = 3$.** The 3-neighborhood of gene g at genomes G_1 and G_2 are $\{d, e, f, h, i, j\}$ and $\{f, k, a, c, i, e\}$ respectively. Since their intersection is the set $\{f, i, e\}$ of size 3, we get that the SI of gene g is 3. Since genes x and ℓ exist only in one genome, their SI is zero. (b) Gene d was transferred from Donor species G_1 to recipient species G_2.

A genome undergoes events of gene gain and loss in which genes are added or removed respectively. These events produce variations over the gene repertoire of the various genomes. In HGT, a gene of a donor genome is inserted at some position in another, recipient, genome. If there exists an ancestral homolog to that acquired gene, and given sufficient evolutionary time, one of the following will occur. Either one of the two copies will be removed (i.e. a "gene loss" to one of the copies), or one of the two copies will mutate (e.g. become a pseudogene), so that the two copies will not be identical (at

least functionally). Under our framework, the new gene will nearly always be integrated between two genes (as genes are integral units), unless integrated at the edge of a linear chromosome (see Figure 2(b)). Consequently, the chance that the gene maintains in the recipient genome its old k-neighborhood (that is, the k-neighborhood from the donor), or even part of it, is very small (assuming k is significantly smaller than the genome size), since such an event means that the gene must be inserted at the same location it has in the donor genome (an event of homologous recombination [2]).

Therefore, low SI of a specific gene, suggests the possibility that the gene has undergone HGT. We note that low SI can be an evidence of other gene recombination phenomena such as duplication or translocation. However as already mentioned, the latter are very infrequent, and we can ignore them in our analysis.

The discussion above focused on a single gene. However, on a whole genome level, the more HGT a genome undergoes, the lesser its similarity, in terms of gene order, to related genomes. Hence, we expect that genomes which diverged long ago will exhibit small synteny to each other. In this case, we cannot use the low SI of a certain gene as an indication for HGT of that gene, since most genes will exhibit quite low SI. However, we can look at the actual *values* of the SI and use them to measure distances between the genomes exposed to high HGT activity. Note that common neighborhoods are defined only to genes that coexist in the two genomes. As genomes become more divergent from each other, such common genes become more rare due to gene loss events. However, recall that we defined SI as zero for a gene found in only a single genome.

We seek a measure that will consider the SI of all genes in the genome. We therefore take the *average* k-SI between the two genomes, denoted as $\overline{SI}$. Also, for the sake of standardization, we normalize by the size of the neighborhood $2k$, so we obtain values in the interval $[0, 1]$. While the two above measures - SI of a single gene and the average SI - were used to either detect a HGT of a gene [1] or define a similarity measure between a set of genomes [3], here we use that measure to distinguish between groups of genes that

presumably exhibit different levels of mobility. Therefore, we extend the latter form. Instead of applying the $\overline{SI}$ over the whole gene set, we apply it separately either only to the pseudogenes set, or only to the non-pseudogenes set. That is, when applying to a gene, pseudogene or non-pseudogene, we compute its SI with respect to its *whole* neighborhood (pseudogenes and non-pseudogene, with $k = 10$). However, the average is taken only for genes of one of the sets each time. We can then compare, quantitatively, these two averages.

3 Gene Duplication Assessment

Our synteny analysis in the main manuscript is based on the assumption that low SI is an outcome of HGT. Low SI however can result from events of duplication as well, questioning the validity of our conclusions. Here we describe the procedure we took to discard the possibility of bogus low SI generated by gene duplications.

Therefore, in order to confirm our finding above, we conducted the following analysis. We asked whether PG tend to be more associated with gene duplications than NPG. We reasoned that duplicated genes could be more prevalent in PG as these could leave one functional copy of the genes. Our analysis was conducted by computing all-against-all alignments of PG and NPG using TBLASTX (translated nucleotide query vs. translated nucleotide subject) alignments, since we have no protein product for pseudogenes. "Duplicates" were considered genes which had alignments covering at least 80% of both subject and query with an e-value $< 1^{-10}$. In contrast to our expectations, we found that the rate of duplications among both PG and NPG is very low and similar among the two groups (Table 1), suggesting that duplications do not play a significant role in the evolution of PG.

	Total	Duplicated	Duplicated (%)
Pseudogenes	1,076	19	1.77%
Coding genes	1,551	23	1.48%
Total	2,627	42	1.60%

Table 1: **Duplication rates in PGs and NPGs.** No significant difference between the two groups was detected.

References

[1] Orit Adato, Noga Ninyo, Uri Gophna, and Sagi Snir. Detecting horizontal gene transfer between closely related taxa. *PLoS Comput Biol*, 11(10):1–23, 10 2015.

[2] Bruce Alberts. *Molecular biology of the cell.* Garland Science, New York, 2002.

[3] Anton Shifman, Noga Ninyo, Uri Gophna, and Sagi Snir. Phylo si: a new genome-wide approach for prokaryotic phylogeny. *Nucleic Acids Research*, 42(4):2391–2404, 2014.
